# Micronutrient deficiency, dietary diversity, and sociodemographic and lifestyle determinants of dietary diversity among pregnant slum-dwelling women in Pune, India

**DOI:** 10.1186/s40795-024-00915-0

**Published:** 2024-07-31

**Authors:** Swapna Deshpande, Rubina Mandlik, Anuradha V. Khadilkar, Jasmin Bhawra, Tarja I. Kinnunen

**Affiliations:** 1 Unit of Health Sciences, Faculty of Social Sciences, Tampere University, Tampere, Finland; 2 Growth and Paediatric Endocrine Department, Hirabai Cowasji Jehangir Medical Research Institute, Pune, Maharashtra India; 3 Interdisciplinary School of Health Sciences, Savitribai Phule Pune University, Pune, Maharashtra India; 4 CHANGE Research Lab, School of Occupational and Public Health, Toronto Metropolitan University, Toronto, Canada

**Keywords:** Micronutrient deficiency, Dietary diversity, Diet quality, Pregnant slum-dwelling women, Urban health, India

## Abstract

**Background:**

Increasing dietary diversity is a sustainable solution to combat micronutrient deficiencies. Given the large slum population in urban India, double burden of malnutrition, nutritional transition among slum-dwellers, and limited studies focusing on dietary intake and diversity among pregnant slum-dwellers, this study aimed to 1) describe macro- and micronutrient intakes and compare them with guidelines, 2) describe dietary diversity and intake of unhealthy foods and, 3) investigate the sociodemographic and lifestyle determinants of adequate dietary diversity among pregnant slum-dwellers in Pune, Maharashtra, India.

**Methods:**

This study presents cross-sectional data of 454 pregnant slum-dwelling women completing mid-pregnancy visit collected from a larger cohort study. Sociodemographic and lifestyle data were collected at baseline (< 12 weeks gestation). Dietary data (24-h dietary recall) were collected in mid-pregnancy (23 ± 2 weeks). Nutrient intakes were compared with the Estimated Average Requirements (EAR) for pregnant Indian women. Dietary diversity score (DDS, range 0–10) and unhealthy food (sweet snacks, sweet beverages, fried and salty food) group score (range 0–3) were calculated as per FAO guidelines. Multivariate logistic regression was conducted to examine determinants of adequate dietary diversity (DDS ≥ 5).

**Results:**

The average age of women was 25 (4.5) years. The median (Q_1_, Q_3_) total energy and protein intakes were 1771 (1456, 2185) kcal/d and 44.7 (34.7, 55.0) g/d, respectively. Total energy and protein were consumed as per EAR by 37% and 54% of women, respectively. Forty percent of women exceeded the recommended energy intake from carbohydrates. Diets of slum-dwelling women were lacking in multiple micronutrients (especially iron, zinc, riboflavin, thiamine, folate). The mean DDS was 4.2 ± 1.2 and 36.5% of the women had DDS ≥ 5. All women consumed mainly cereal-based starchy staples; 80% consumed pulses and legumes, and 60% consumed other vegetables. Fifty-nine percent of women consumed ≥ 2 unhealthy food groups. Higher educational and occupational status of the primary earning members of the family and lower parity were determinants of adequate dietary diversity.

**Conclusion:**

The diets of pregnant slum-dwelling women were lacking in numerous micronutrients. Dietary counselling programs need to be tailored to the socioeconomic backgrounds of pregnant slum-dwelling women and involve their family members to improve reach and effectiveness.

## Background

Micronutrient deficiency is a global public health challenge. It affects human health, and societal well-being by translating into economic losses, especially in low- and middle-income countries (LMICs) including India [1, 2]. Half of the global population with micronutrient deficiencies lives in India [3–5]. Poor diet quality is a common cause of micronutrient deficiency. Increasing food-based dietary diversity is a sustainable and effective strategy to combat it [6, 7]. Dietary diversity is defined as the number of different food groups or foods consumed in a given period, most often the previous day or week [8]. Diverse diets provide a balanced and adequate combination of macronutrients, essential micronutrients, and other food-based substances such as dietary fibres, and ensure nutrient adequacy [9].

Maternal nutrition has both short and long-term impacts on maternal and child health. Micronutrient deficiencies during pregnancy can cause adverse outcomes such as anaemia and hypertension in the mother [10], and neural tube defects in babies, and it can increase the risk of delivering low birth weight and small-for-gestation age infants [11–13]. Low-quality maternal diets have serious implications on child health and survival within the first 1000 days of a child’s life [4, 12]. The malnutrition paradox is a significant issue in India, and it is also observed among the urban poor. This paradox involves the persistence of underweight conditions coexisting with an increasing prevalence of overweight and obesity[14, 15]. A study based on the national survey in India reported a similar prevalence (~ 21%) of underweight (body mass index, BMI < 18.5 kg/m^2^) and obesity (BMI > 25 kg/m^2^) among urban poor mothers [16]. Maternal underweight and overweight along with micronutrient deficiencies exacerbate the risk of adverse pregnancy outcomes [2, 4, 17].

Due to the rapidly growing economy, India is steadily urbanising [18], and 36% Indians live in urban areas [19]. This has created an enormous infrastructural burden and has led to a significant increase in people living in slums. According to the United Nations Human Settlements Programme, a slum is “a group of individuals who live under the same roof, and that lack one or more of the following conditions: access to improved water, access to improved sanitation, sufficient living space, the durability of housing, and secure tenure” [20]. According to census 2011, approximately 17% of urban Indians reside in slums. Slum dwellers face social, economic, and health-related challenges due to unstable living conditions (inadequate access to safe water, sanitation, secure residential status) that impact their ability to adopt healthy dietary patterns. Studies have shown that economic constraints magnify the problem of limited access to micronutrient-rich healthy foods [21]. The food environment in slums provides easy access to energy-dense snacks, beverages, and processed and packed foods which makes slum-dwellers susceptible to overweight, obesity, and related non-communicable diseases. It is crucial to specifically address the health of the large population of slum dwellers. The ongoing reliance on urban average data obscures intra-urban disparities, leading to suboptimal success in India's flagship nutrition initiatives. [16].

In an effort to achieve SDGs 2 (zero hunger), 3 (good health and well-being), and 11 (wellbeing of cities), it is critical to design interventions focusing on maternal and child health among slum dwellers, who are the most vulnerable in the urban context. There are very few longitudinal studies on nutrition among slum-dwelling pregnant women in India. Many studies focusing on dietary diversity among pregnant Indian mothers are rural-centric [22–25]. Very few studies have collected information on unhealthy food groups which account for the rising nutrition transition in urban India. With this gap in mind, this study aims to 1) describe macro- and micronutrient intakes and compare them with the national guidelines in India, 2) describe dietary diversity and unhealthy food intake and 3) investigate the sociodemographic and lifestyle determinants of adequate dietary diversity among pregnant slum dwellers in Pune, Maharashtra, India. The findings of this study may enable designing effective dietary interventions among pregnant slum-dwelling women.

## Methods

### Study design and study population

Pune is one of the fastest-growing megacities in Maharashtra, Western India, with an estimated population of 4,307,000 [26]. The growing economic opportunities in Pune has prompted migration from rural regions, leading to an increasing number of individuals residing in slums within the city (> 1.2 million residents) [27].

Public antenatal care (ANC) clinics are major centres in the city for slum-dwelling women to avail skilled care and birthing facilities free of cost. Mother and Infant (MAI) is an ANC-based observational cohort study among pregnant slum-dwelling women in urban Pune. The public ANC clinic was selected using convenience sampling. It is located in the geographical proximity of large, registered slums near Bhavani Peth, Dhole Patil Road, and Sangamwadi area of Pune. These are the largest establishments of the slums in Pune city and each of them consists of an established setting of 15,000–20000 households with a population of around 80,000 to 100,000 [27]. The women were registered at the ANC and visited the facility for routine ANC checkups and delivery. The research field staff explained the study to the women attending these clinics. Women who were willing to participate in the study were screened for eligibility. Eligibility criteria included a) willingness to participate in the study and ability to give informed consent, b) residence in a registered slum, c) gestational age less than 12 weeks, d) singleton low-risk pregnancy, e) absence of major health issues (hypertension, diabetes mellitus, tuberculosis, kidney disease) that require intensive hospital follow-up, and f) intention to attend the same ANC clinic and to deliver in the same health facility or in the same city. The length of gestation was calculated by self-reported date of the last menstrual period in the healthcare record and confirmed using the ultrasound reports available at the clinic. Residential addresses were collected during the screening process by field workers who were well acquainted with the slums that participants lived in. The study participants were contacted a week prior to their scheduled follow-up visits by the field workers.

**Fig. 1:**
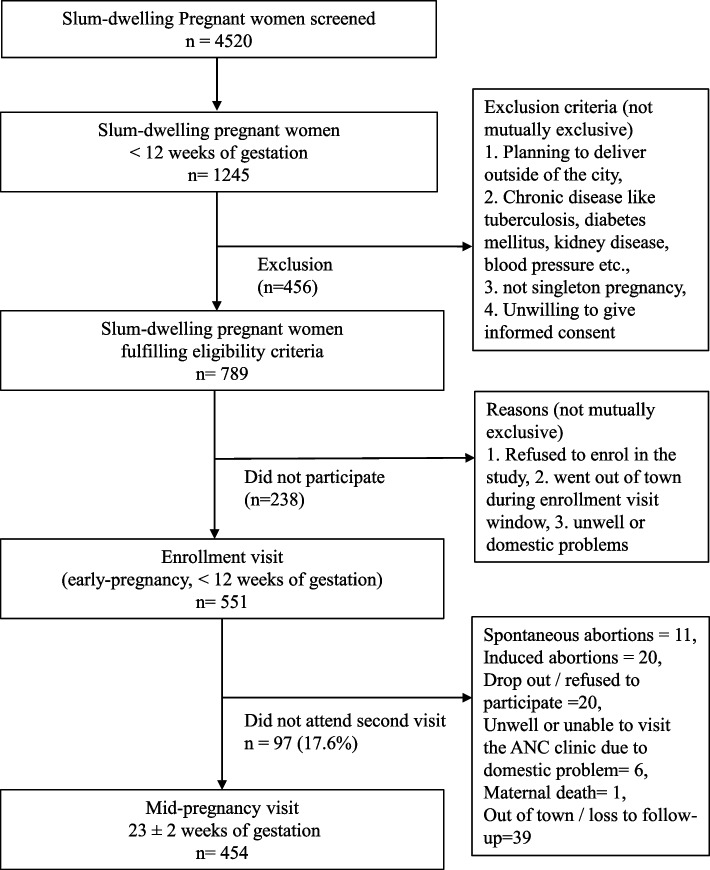
Flowchart of the study sample of pregnant slum-dwelling women who completed mid-pregnancy visit, Pune, India, *n* = 454

Of the 4520 women screened, 789 women (17.5%) were eligible, and 551 women (70% of eligible) were enrolled in the study. The mid-pregnancy visit was completed by 454 (82%) of 551 enrolled women (Fig. 1). The present study uses cross-sectional data from the larger cohort study, which includes 454 pregnant slum-dwelling women completing the mid-pregnancy visit. The study commenced in January 2021, and recruitment was completed in March 2023, and the mid-pregnancy follow-up visits in July 2023.

### Ethics approval and consent

Written informed consent was obtained from the women after they were explained and had understood the participant information sheet. If an eligible woman was illiterate, a thumb impression was taken from her after ensuring that she had understood and had also explicitly stated her consent verbally; a literate impartial witness signed the consent form on her behalf. Ethics approvals were obtained from the Ethics Committee Jehangir Clinical Development Centre Pvt Ltd. (EC Registration No: ECR/352/lnst/MH/2013/RR-19) and the Ethics Committee of the Tampere Region. All methods were performed in accordance with the relevant guidelines and regulations.

### Data collection in early and mid-pregnancy

Trained research staff collected data at the two time points during pregnancy: enrollment in the study before 12 weeks of gestation (‘early-pregnancy’/baseline visit), and at 23 ± 2 weeks of gestation (‘mid-pregnancy’). The study participants were contacted a week prior to their scheduled follow-up visits by the field workers. In the current study, we present socio-economic and lifestyle data from the baseline visit and dietary data from mid-pregnancy. Data from early pregnancy were not considered representative of the actual dietary intakes due to reduced dietary intake as a result of nausea and vomiting [28]. In the present study, over 70% of women reported either nausea or vomiting in early pregnancy, and ~ 20% of women reported nausea or vomiting in mid-pregnancy. Hence, we have presented dietary intakes in mid-pregnancy as a proxy for dietary intakes during pregnancy in this study. Sociodemographic and lifestyle information, and obstetric and gynaecological history were collected in a face‐to‐face interview at the baseline visit. Sociodemographic information included information on age of the participant, education and occupation/working status of the primary earning family member and the participant, monthly household income (total income of all family members in the house), and religion of the participant. Educational attainment was categorised as 1) less than or equal to middle school certificate (completed 8 years of education), 2) middle school to higher secondary certificate (completed 12 years of education), and 3) graduate degree or higher, or professional diploma [29]. Occupation of the primary earning family member was categorised as 1) unemployed, unskilled, or semi-skilled, 2) skilled, or arithmetic skill, and 3) semi-professional and professional [29]. Working status of the participant was categorised as homemakers or working. Lifestyle information included data on substance abuse: tobacco chewing, mishri (a form of smokeless tobacco), gutka (a preparation of crushed tobacco, areca nut, slaked lime, catechu, and flavouring agent), bidi (a thin cigarette filled with tobacco flakes wrapped in a tendu-leaf)/cigarette, and alcohol, and if the participant exhibited pica, i.e., the craving and consumption of non-food items such as ice, chalk, soil, etc. Information related to pregnancy-related complaints (nausea, vomiting, giddiness, heartburn, etc.) was collected at both visits.

Regarding anthropometric measurements, height was measured to the nearest cm using a portable stadiometer (SECA model 213, Hamburg, Germany) during the baseline visit. Weight was measured to the nearest 100 g using calibrated weighing scales at both visits. The baseline BMI (a proxy for pre-pregnancy BMI) was calculated based on the first measured weight during early pregnancy and classified according to Asian-specific BMI cut-off points [30].

### Nutrient intake, dietary diversity, and unhealthy food group consumption

The main outcomes of this study were macro- and micronutrient intakes, dietary diversity score (DDS) and unhealthy food group score. A trained nutritionist collected data on dietary intake over three non-consecutive days (two weekdays and one weekend day) using the multiple-pass 24-h recall method. One weekend day was included in the recall to accommodate the variation in the dietary intake that the women might have over the weekend. Standard serving bowls, plates, spoons, and models of foods like roti (Indian flatbread) were used to obtain an accurate estimate of portion sizes. Nutrient analysis software (C-Diet) [31], which uses the Indian food composition tables, a cooked food database, and the USDA raw food database [31], was used to calculate dietary macro- and micronutrient intake. The nutrient intakes reported are an average of the nutrients consumed over the three days.

The DDS in mid-pregnancy was calculated using the Minimum Dietary Diversity for Women (MDD-W) indicator [9]. This simple food-based indicator measures the diet quality based on the food items consumed (> 15 g) during the previous day using 24-h diet recall data (Table 3). Ten food groups were considered for calculating the DDS as predetermined by the Food and Agricultural Organization (FAO) [9]. The DDS ranges between 0–10 based on the number of food groups consumed. Women achieving DDS ≥ 5 were categorised as having ‘adequate dietary diversity’ and those with DDS < 5 were categorised as having ‘inadequate dietary diversity’.

As per the FAO guidelines, the following three food groups were considered for constructing unhealthy food group score: 1) Fried and salty snacks and food, 2) sweet snacks and foods, and 3) sweet beverages (Table 3). The score ranged from 0 to 3 and women with scores ≥ 2 were categorised as ‘unhealthy food eaters’. Unhealthy food groups were not considered in the construction of DDS [9].

### Data analysis and statistical methods

Data are presented as the mean and standard deviation (SD) and median (Q_1_, Q_3_) values for continuous variables and number (%) for categorical variables. We have described the absolute macro- and micronutrient intakes and compared them with the Estimated Average Requirements (EAR) for pregnant Indian women [32]. We have calculated the percent of energy (E%) obtained from macronutrients and compared them with the Acceptable Macronutrient Distribution Range for each macronutrient [32].

The median macro- and micronutrient intakes, in mid-pregnancy were presented for the inadequate and adequate DDS groups. The macro- and micronutrient intakes were adjusted for total energy intake using the residual methods of Willet and Stampfer [33]. Implausible and extreme nutrient values beyond 3 SD were truncated to 3 SD to remove outliers (27 datapoints).

We compared nutrient adequacy (as a summary measure of nutrient intakes) in inadequate and adequate dietary diversity groups by calculating nutrient adequacy ratio (NAR) and mean adequacy ratio (MAR) [34]. We derived NAR using individual’s nutrient intake. NAR was calculated for 12 nutrients (carbohydrate, protein, vitamin A, vitamin B_1_, vitamin B_2_, vitamin B_3_, vitamin B_9_, vitamin C, iron, calcium, zinc, and magnesium). NAR was calculated as ratio of participant’s nutrient intake and EAR (reference nutrient intake). NAR values were truncated at 1 to prevent the compensation of a nutrient with NAR greater than 1 for a nutrient with a lower NAR. MAR was calculated as the average NARs of 12 nutrients. The MAR was reported on the scale of 0 to 1 [34] and compared between adequate and adequate dietary diversity groups using two sample t-test for statistical significance test.

Bar charts were used to present the proportions of women consuming individual DDS food groups in the inadequate and adequate dietary diversity groups. The proportion of unhealthy food eaters were compared between these groups using the chi-square test. Univariate and multivariate logistic regression analyses were performed to investigate the determinants of adequate dietary diversity and the results were reported using crude and adjusted odds ratio (aOR) with 95% confidence intervals (CI). The following explanatory variables were used: participant’s education, working status, use of mishri, exhibition of pica, and BMI status at baseline, as well the education and occupation of the primary earning family member, and monthly household income. In addition to all the above variables, the multivariate models were adjusted for 1) age and religion, and 2) age, religion, and energy intake as confounding factors. The model adequacy of regression analyses was checked using goodness of fit measures.

The potential impact of seasonality on dietary intake was analysed. The seasonality variable was constructed using the month of interviewing women for their dietary intake. Three seasons were considered as per the western part of India: 1) winter: November to February 2) summer: March to June 3) monsoon: July to October. We compared macro- and micronutrient intakes in three seasons. We also added the seasonality variable in the logistic regression analysis to check the robustness of the results.

All statistical analyses were performed using SAS version 9.4 and R 4.0.3.

## Results

Table 1 presents baseline (early-pregnancy) demographic and lifestyle behavioural characteristics of 454 women. Their mean age was 25 (4.5) years (range 16.2–41.0 years), 61% were Hindus, and 56% were nulliparous. One-third of the women had completed at least 8 years of education, most of the women were homemakers, and nearly all women were non-vegetarians. Similar percentages of women (~ 25%) were underweight and overweight, and 11% women were obese. Nearly 70% of the primary earning members were engaged in skilled jobs (carpentry, painting, masonry, driving, etc.) or arithmetic skilled jobs (petty shopkeeping, supervision of labourers). The pregnant women who enrolled in the study but did not attend the mid-pregnancy visit (*n* = 97) had similar age, education, occupation, weight, height, and total energy intake as compared to those who completed the mid-pregnancy visit (data not shown).

**Table 1:** Baseline sociodemographic and lifestyle characteristics of pregnant slum-dwelling women in Pune, India (*n* = 454)

Characteristics of the study participants		Mean (SD)	Median (Q_1_, Q_3_)
Age (years)		25 (4.5)	24 (21, 27)
Weight (kg)		51.6 (11.2)	50.0 (43.6, 57.0)
Height (cm)		153.3 (5.8)	153.2 (149.4, 157.0)
BMI (kg/m^2^)		21.9 (4.5)	21.1 (18.6, 24.7)
Monthly family income (INR)		25,107 (18,542)	20,000 (14,000, 30,000)
BMI categories (WHO criteria, Asians), n (%)			
Underweight	< 18.5 kg/m^2^	112 (24.7%)	
Normal weight	18.5–23.0 kg/m^2^	178 (39.2%)	
Overweight	23.0–27.5 kg/m^2^	117 (25.8%)	
Obese	> 27.5 kg/m^2^	47 (10.4%)	
Marital status, n (%)	Married	453 (99.8%)	
	Divorced	1 (0.2%)	
Participant educational attainment, n (%)	Middle school certification or below	141 (31%)	
	High school to higher secondary certificate	249 (55%)	
	Graduate degree and above or professional diploma	64 (14%)	
Participant’s working status, n (%)	Homemakers	398 (88%)	
	Working	56 (12%)	
Educational attainment of primary earning member in family, n (%)	Middle school certification or below	141 (31%)	
	High school to higher secondary certificate	232 (51%)	
	Graduate degree and above or professional diploma	81 (18%)	
Occupation of primary earning member in family, n (%)	Unemployed, unskilled or semi-skilled workers	100 (22%)	
	Skilled or arithmetic skilled jobs	329 (72%)	
	Semi-professional or professional jobs	25 (6%)	
Religion, n (%)	Hindu	276 (61%)	
	Muslim	152 (33%)	
	Others	26 (6%)	
Food habits, n (%)	*Vegetarian	21 (5%)	
	Ovo- or Non-vegetarian	433 (95%)	
Parity, n (%)	Nulliparous	253 (56%)	
	Primiparous	158 (35%)	
	Multiparous	43 (9%)	
Lifestyle behaviour, n (%)	Tobacco chewing (yes)	1 (0.01%)	
	Mishri (yes)	28 (6%)	
	Gutka (yes)	0 (0)	
	Bidi or cigarette smoking (yes)	1 (0.01%)	
	Alcohol consumption (yes)	0 (0)	
	Pica (yes)	33 (7%)	

*Legend*: The values are mean (SD) and median (Q1, Q3) or n (%). BMI: body mass index. Middle school certificate (8 years of education), higher secondary certificate (12 years of education). Primary earning members were husbands or in-laws of the pregnant women. Vegetarian category includes all lacto-vegetarian women

Table 2 presents the average dietary macro- and micronutrient intakes and DDSs of the women. Total absolute amounts of energy and protein as per EAR were consumed by only 37% and 54% of women, respectively. The average percentages of energy obtained from carbohydrates, fats, and proteins were 63%, 27%, and 10%, respectively. More than one-third (39%) of women exceeded the recommended energy intake (45–65 E%) from carbohydrates. Majority (> 90%) of the women met the recommended energy intake (20–35 E%) from fat, whereas energy intake from protein was within the recommended range (5–15 E%) for all women. The diets were devoid of most micronutrients; 41% of women consumed niacin below the EAR, 85% had vitamin A intakes below the EAR, and the dietary intakes of iron, zinc, riboflavin, and folate were below the EAR for nearly all (98%) women. The macro- and micronutrient intakes did not vary in the three seasons (p > 0.05, for all) (data not shown).

**Table 2:** Macro and micronutrient profile and dietary diversity among pregnant slum-dwelling women in Pune, India (*n* = 454)

	Mean (SD)	Median (Q_1_, Q_3_)	EAR	Percentages of women consuming below EAR (95% CI)
Macronutrients				
Total energy (kcal/d)	1834 (524)	1771 (1456, 2185)	2010	64.3 (59.9, 68.7)
Carbohydrate (g/d)	286 (77)	281 (231, 342)	130	2.2 (0.9, 3.6)
Proteins (g/d)	46.5 (16.6)	44.7 (34.7, 55.0)	44.0	46.5 (41.8, 51.2)
Fat (g/d)	55.9 (22.0)	52.7 (40.0, 69.0)	-	
Fibre (g/d)	22.3 (7.8)	21.5 (16.7, 27.0)	-	
% energy from carbohydrates^a^	62.9 (6.2)	63.4 (58.6, 67.3)	45–65%	0.7 (0.1, 1.9)
% energy from fat^a^	27.0 (5.1)	26.7 (23.5, 30.4)	20–35%	8.4 (6.0, 11.3)
% energy from proteins^a^	10.1 (1.7)	9.8 (8.9, 11.0)	5–15%	0
Micronutrients				
Vitamins				
Vitamin A (µg/d)^b^	267.7 (170.9)	224.6 (158.1, 331.6)	406.0	85.0 (81.7, 88.3)
Vitamin B_1_ (Thiamine) (mg/d)	0.75 (0.38)	0.67 (0.50, 0.90)	1.6	97.4 (95.9, 98.8)
Vitamin B_2_ (Riboflavin) (mg/d)	0.63 (0.47)	0.50 (0.34, 0.78)	2.3	98.7 (97.6, 99.7)
Vitamin B_3_ (Niacin) (mg/d)	14.7 (9.2)	12.3 (9.0, 17.0)	11.0	40.5 (36.0, 45.1)
Vitamin B_9_ (Folate) (µg/d)	101.0 (62.3)	86.9(65.7, 118.0)	480.0	99.8 (99.1, 100.0)
Vitamin C (mg/d)	45.5 (24.3)	39.3 (28.3, 57.7)	65.0	82.6 (78.8, 86.0)
Minerals				
Iron (mg/d)	9.9 (3.9)	9.4 (7.1, 12.3)	21.0	99.3 (98.6, 100.0)
Calcium (mg/d)	496.8 (250.0)	445.3 (307.7, 637.0)	800.0	88.3 (85.4, 91.3)
Zinc (mg/d)	5.4 (2.1)	5.1 (3.9, 6.5)	12.0	99.1 (98.3, 99.9)
Magnesium (mg/d)	363.5 (132.4)	346.5 (268.7, 439.0)	370.0	57.5 (52.9, 62.0)
				Percentages of women with inadequate dietary diversity score (95% CI)
Diet diversity score	4.2 (1.19)	4 (3, 5)	5	63.4 (58.9, 67.7)
Unhealthy food group score^c^, n(%)				
0	37 (8.1%)			
1	151 (33.3%)			
2 / 3	266 (58.6%)			

*Legend*: Estimated Average Requirement (EAR) levels and ^a^Acceptable Macronutrient Distribution Range are based on nutrient requirement of pregnant women (*Revised Short Summary Report -2024, ICMR NIN Expert Group on Nutrient requirements for Indians, Recommended Dietary Allowances (RDA) and Estimated Average Requirements (EAR)-2020*)

^b^retinol activity equivalents (RAE)

^c^n (%) of women consuming a number of unhealthy food groups. Nutrient data are based on the 24-h recall of three days. The nutrient data do not include nutrients consumed from supplements. No EAR for fat and fibre intake. Unhealthy food groups are a) fried and salty snacks, b) sweet food, and c) sweet beverages

**Fig. 2:**
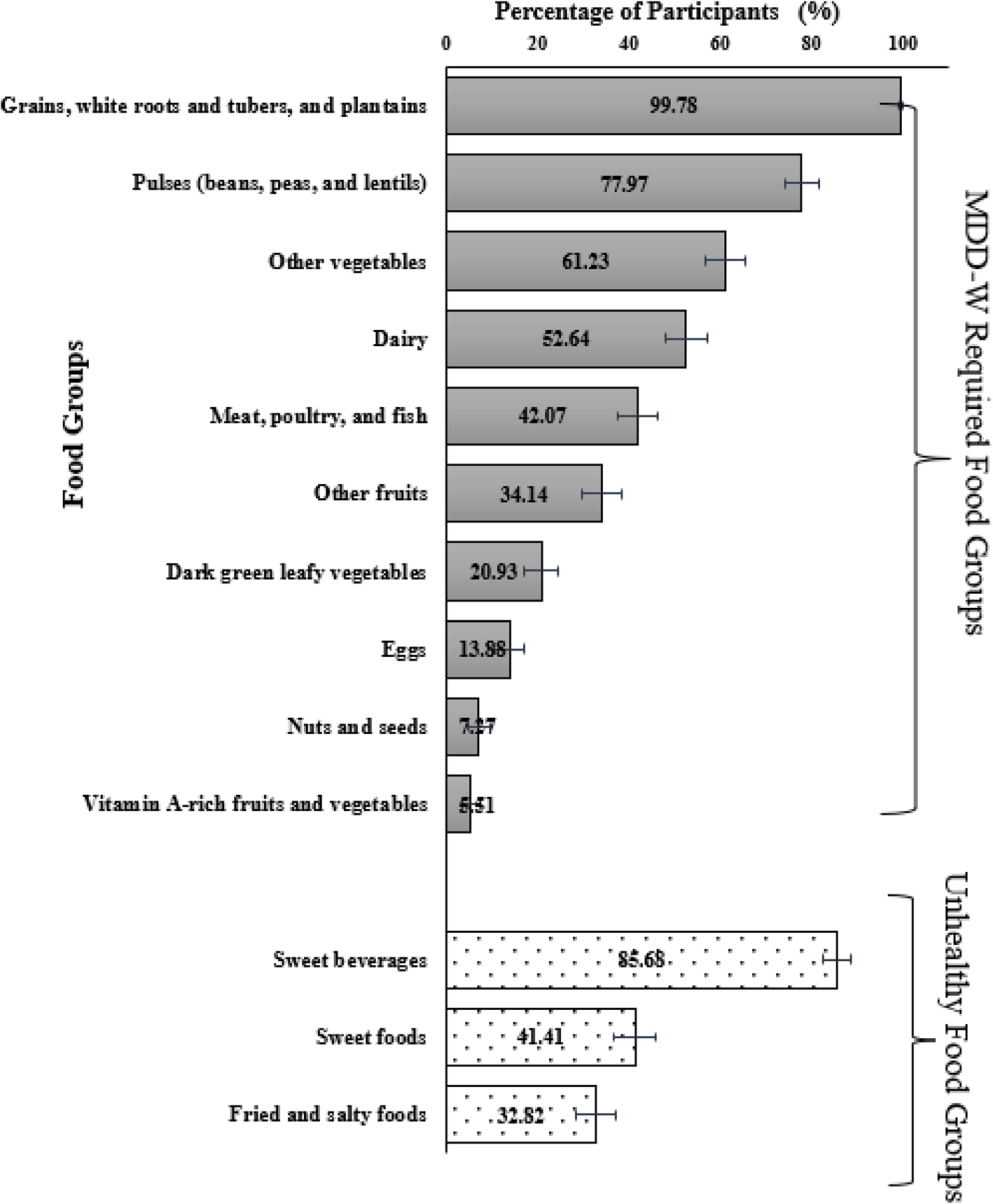
Participants consuming MDD-W required food groups and unhealthy food groups. Legend: Dietary data were collected among 454 slum-dwelling women in mid-pregnancy (23 ± 2 weeks) visit, Pune, India. MDD-W: Minimum Dietary Diversity for Women. The error bars represent 95% confidence interval

The mean DDS was 4.2 (1.2), and 63% of women (*n* = 288) had inadequate dietary diversity in mid-pregnancy. Only 50% of women were eating at least 4 food groups (Q_1_, Q_3_, (3, 5)). Table 3 presents examples of frequently consumed foods in diet diversity score groups and unhealthy food groups. All women consumed starchy staples (food made from cereals, grains, tubers, white roots, and plantains); approximately 80% consumed pulses and legumes, and 60% consumed other vegetables. Only half of the women consumed milk and/or milk products, and 21% consumed dark leafy vegetables. Very few women (< 10%) consumed nuts and seeds and vitamin A-rich fruits and vegetables (Fig. 2). Nearly 59% (*n* = 266) of women were categorised as unhealthy food eaters. Among the unhealthy food groups, 86% of women consumed sweet beverages owing to frequent consumption of tea with sugar, and 41% consumed sweet snacks or foods, and 33% women consumed fried and salty snacks (Fig. 2). The unhealthy food eaters and the rest had similar baseline sociodemographic characteristics, except pica eating. The unhealthy food eaters had higher percentage of pica eating compared to the rest (9.5% vs. 3.2%, *p* = 0.017).

**Table 3:** Examples of frequently consumed foods in diet diversity score groups and unhealthy food groups

Diet diversity food groups	Frequently consumed food items by the study participants
Grains, white roots and tubers, and plantains	chapati / roti (flattened unleavened bread of whole wheat flour), bread, bhakari (flattened unleavened bread of millet flour), rice, pulao (rice cooked with spices and vegetables and / or chicken, meat), biryani (rice cooked with spices and curried vegetables and / or chicken, meat), idli / dosa (fermented rice and pulse preparations), pohe (savoury flattened rice), potato vegetable (curried / fried / boiled)
Pulses (beans, peas, or lentils)	dal (curried pulse preparation), pithale (semi-liquid bengal gram preparation), misal (spicy sprouts curry), chickpeas / kidney beans / black eyed peas curry, medu vada (fermented deep fried pulse batter shaped as doughnuts), idli / dosa (fermented rice and pulse preparation)
Nuts and seeds	peanuts, almonds, walnuts, cashews
Dairy (milk and dairy foods)	milk (cow, buffalo), curd, buttermilk, paneer (cottage cheese)
Meat, poultry, and fish	poultry / meat curry, curried /fried fish, poultry / meat biryani, kheema (minced meat preparation)
Eggs	eggs (hen) omelette, scrambled, curried, boiled, fried
Dark green leafy vegetables	savoury curried or dry preparations of spinach, fenugreek, dill, spring onion leaves, green and red amaranthus
Vitamin A-rich fruits and vegetables	pumpkin (savoury curried or dry preparation), carrot (salad), mango (seasonal)
Other vegetables	savoury curried or dry preparations of ladies finger / okra, cabbage, cauliflower, brinjal / aubergine, tomato, cluster beans, onion, cucumber, capsicum
Other fruits	apple, banana, guava, sapodilla, sweet lime, watermelon, pomegranate
Unhealthy food group	
Fried and salty foods	potato vada (deep fried potato patty), bhaji (onion or potato fritters), samosa, banana chips, potato wafers, khari pattice (spicy vegetable filled puff pastry), gobi manchurian (deep fried cauliflower coated with corn flour), packaged cornpuff sticks (crunchy puffcorn snacks made up of rice, lentil and corn)
Sweet foods	sweet biscuits, jam and / or cream-filled biscuits, candies, chocolates, ice-cream, cream roll (cream filled puff pastry rolls), jalebi (deep fried preparation of bengal gram flour soaked in sugar syrup), gulab jamun (deep fried balls made from refined wheat flour and evaporated milk balls and soaked in sugar syrup)
Sweet beverages	tea, coffee, fruit milkshake, popular tetra pack mango flavoured drink, carbonated beverages, lime juice with sugar

*Legend*: Most of the fried and salty snacks, sweet beverages and sweet snacks are packed snacks and bought from the local vendors or petty shops. Tea with sugar (median 1.5 tablespoons per 200 ml of tea)

### Characteristics of the diets of women with adequate dietary diversity and its sociodemographic and lifestyle determinants

**Fig. 3:**
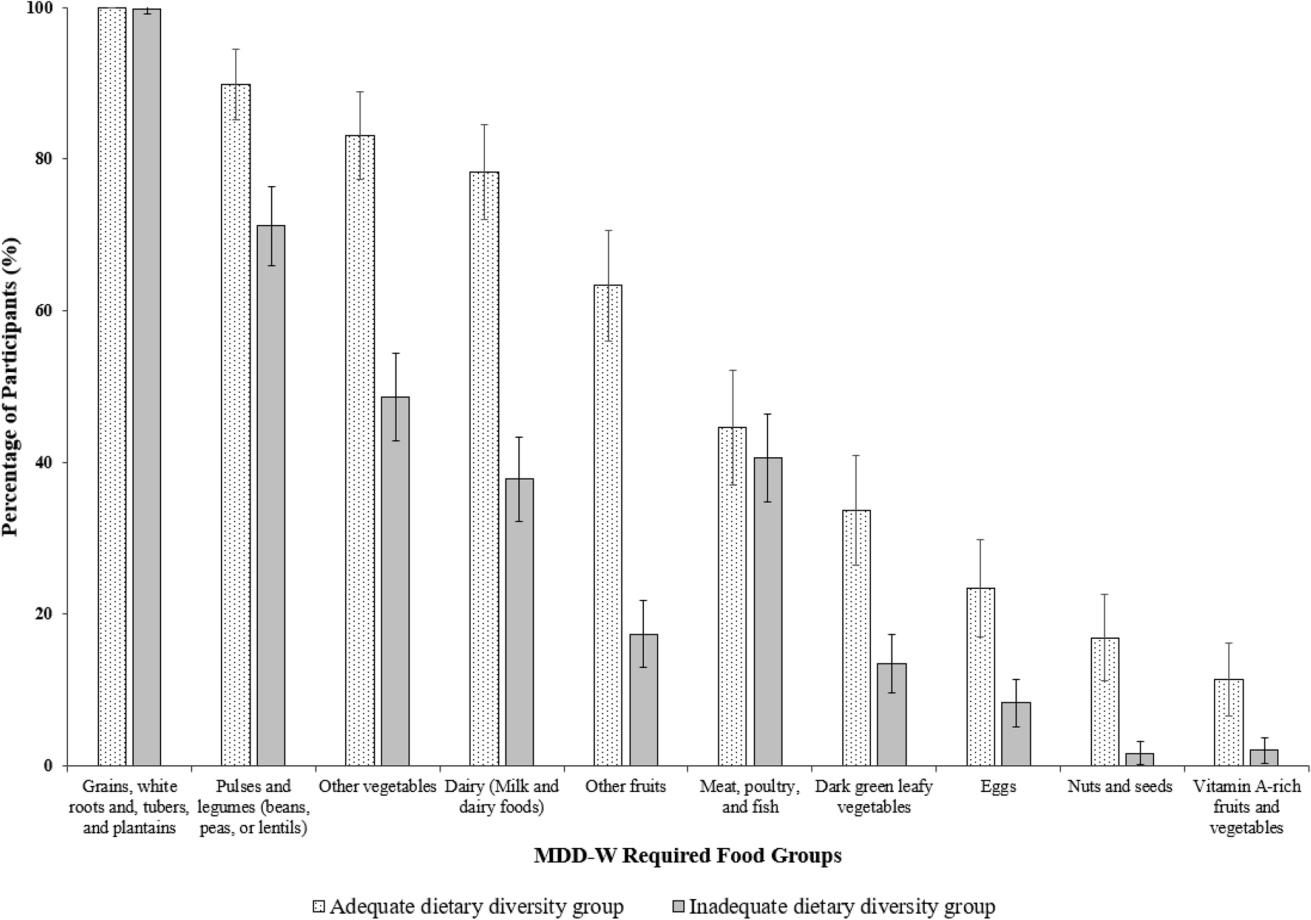
Percentage of women consuming MDD-W required food groups in adequate and inadequate dietary diversity groups. Legend: Dietary data were collected among 454 slum-dwelling women in mid-pregnancy (23 ± 2 weeks) visit, Pune, India. MDD-W: Minimum Dietary Diversity for Women. The error bars represent 95% confidence interval

The adequate dietary diversity group had higher median intakes of total energy, protein, and fibre, similar fat intake, and lower carbohydrate intake compared to the inadequate dietary diversity group. The median micronutrient intakes are presented in inadequate and adequate dietary diversity groups (Table 4). The mean MAR was higher in the adequate dietary diversity group compared to the other group (0.66 (SD, 0.15) vs. 0.56 (SD 0.14), p < 0.001). The percentages of women consuming pulses, other vegetables, milk and milk products, other fruits, dark green leafy vegetables, and eggs were higher in the adequate dietary diversity group than in the inadequate dietary diversity group (Fig. 3). The percentage of women consuming unhealthy foods was higher in the inadequate dietary diversity group than in the adequate dietary diversity group, though not statistically significant (63% vs. 52%, *p* = 0.151) (Table 4). The percentage of women consuming sweet beverages / sweet snacks and foods was higher in inadequate dietary diversity group compared to the other group (93% vs. 81%, *p* = 0.04). However, the percentage of women consuming fried and salty snacks and foods were similar in both groups (33% vs. 32%, *p* = 0.57).

**Table 4:** Nutrient intakes in inadequate and adequate dietary diversity groups among pregnant slum-dwelling women

	Inadequate dietary diversity	Adequate dietary diversity
	*n* = 288 (63.4%)	*n* = 166 (36.6%)
Macronutrients		
Total energy (kcal/d)	1724 (1410, 2056)	1966 (1567, 2406)
Carbohydrates (g/d)	291 (271, 310)	270 (261, 288)
Proteins (g/d)	39.7 (35.8, 43.8)	45.4 (40.2, 49.3)
Fats (g/d)	52.2 (45.0, 58.3)	53.4 (45.1, 57.9)
Fibre (g/d)	19.7 (17.1, 24.0)	22.0 (19.2, 25.2)
Micronutrients		
Vitamins		
Vitamin A (µg/d)	212.0 (163.5, 296.8)	259.3 (182.5, 351.0)
Vitamin B_1_ (Thiamine) (mg/d)	0.7 (0.6, 0.8)	0.9 (0.7, 1.0)
Vitamin B_2_ (Riboflavin) (mg/d)	0.5 (0.4, 0.6)	0.7 (0.5, 1.0)
Vitamin B_3_ (Niacin) (mg/d)	12.0 (10.8, 14.9)	14.1 (11.6, 17.8)
Vitamin B_9_ (Folate) (µg/d)	86.1(70.8, 104.6)	103.7 (83.0, 125.7)
Vitamin C (mg/d)	39.0 (30.5, 50.3)	45.7 (35.0, 60.7)
Minerals		
Iron (mg/d)	9.3 (8.3, 11.0)	10.1 (8.6, 11.8)
Calcium (mg/d)	435.4 (350.2, 561.4)	525.0 (414.5, 656.0)
Zinc (mg/d)	5.2 (4.5, 6.1)	5.4 (4.5, 6.4)
Magnesium (mg/d)	347.8 (309.7, 384.3)	382.6 (328.7, 419.6)
Unhealthy food eaters n(%)	180 (63%)	86 (52%)

*Legend*: The values presented are median (Q_1_, Q_3_) or n (%). Adequate dietary diversity (diet diversity score ≥ 5). Unhealthy food eaters (unhealthy food groups ≥ 2). All macro- and micronutrients are adjusted for total energy intake. The nutrient data do not include nutrients consumed from supplements

When baseline sociodemographic and lifestyle determinants were investigated for possible relationships with adequate dietary diversity, the participants’ own higher education, lower parity, the primary earning member’s higher education and occupation in skilled/arithmetic skilled jobs and in semi-professional/professional jobs, and higher household income were associated with higher likelihood of adequate dietary diversity in the univariate analyses (crude OR, Table 5). Education of the primary earning members in the high school to higher secondary category and graduate degree and above category, the primary earning members’ occupation in skilled/arithmetic skilled jobs and in semi-professional/professional jobs, and lower parity of the participants were the significant determinants of adequate dietary diversity, independent of all other variables in the multivariate regression analysis (Table 5). The determinants of adequate dietary diversity remained unchanged when the multivariate model was further adjusted for energy intake. However, the relationship between the education of the primary earning members and adequate dietary diversity got attenuated (Table 5). These results did not change even after adjusting the model for seasonality.

**Table 5:** Baseline sociodemographic and lifestyle determinants of adequate dietary diversity in mid-pregnancy among pregnant slum-dwelling women

			Adequate dietary diversity					
covariates	Categories	n	Crude odds ratio (95% CI), *p* value		^$^ Adjusted odds ratio (95% CI), *p* value		^$$^ Adjusted odds ratio (95% CI), *p* value	
Participant educational attainment	Middle school certification or below	141	1		1		1	
	High school to higher secondary certificate	269	1.65 (1.10 – 2.56)	0.0305	1.11 (0.67–1.82)	0.693	1.15 (0.70 -1.92)	0.579
	Graduate degree/ higher/professional diploma	64	2.45 (1.33–4.56)	0.0049	1.25 (0.60 – 2.60)	0.543	1.31 (0.62 – 2.76)	0.473
Participant’s working status	Homemakers	398	1		1		1	
	Working	56	1.47 (0.83–2.58)	0.183	1.38 (0.74 – 2.54)	0.309	1.46 (0.78 – 2.74)	0.263
Educational attainment of primary earning member in family	Middle school certification / below	141	1		1		1	
	High school to higher secondary certificate	232	2.11 (1.33–3.39)	0.002	1.64 (1.00 – 2.74)	0.050	1.54 (0.97 – 2.60)	0.072
	Graduate degree and above or professional diploma	81	2.92 (1.64 – 5.26)	0.003	1.69 (0.95 – 3.42)	0.091	1.67 (0.93- 3.43)	0.098
Occupation of primary earning member in family	^*^Unskilled or semi-skilled workers	100	1		1		1	
	Skilled or arithmetic skill jobs	329	2.00 (1.21–3.37)	0.008	1.62 (0.97 – 2.86)	0.071	1.55 (0.96 – 2.58)	0.079
	Semi-professional or professional jobs	25	4.75 (1.91 -12.29)	0.001	2.81 (1.12- 7.99)	0.044	2.55 (1.05–7.07)	0.047
Parity	Nulliparous	253	1		1		1	
	Parous	201	0.57 (0.38 -0.83)	0.005	0.63 (0.39 – 0.98)	0.043	0.64 (0.40 -0.99)	0.049
Mishri users	No	426	1		1		1	
	Yes	28	0.56 (0.22 -1.29)	0.195	0.74 (0.27 – 1.82)	0.535	0.70 (0.26 – 1.75)	0.467
Pica behaviour	No	421	1		1		1	
	Yes	33	1.10 (0.51 – 2.30)	0.797	0.93 (0.40 – 2.07)	0.864	1.06 (0.45 -2.38)	0.891
Monthly household income	< 20,000 INR	209	1		1		1	
	≥ 20,000 INR	245	1.55 (1.06–2.29)	0.026	1.16 (0.76 – 1.78)	0.485	1.10 (0.70 -1.66)	0.737
Body mass index categories	Normal weight	178	1		1		1	
	Underweight	112	0.85 (0.52 – 1.38)	0.514	0.95 (0.57 – 1.59)	0.853	0.97 (0.58 – 1.64)	0.919
	Overweight	117	0.79 (0.49- 1.28)	0.351	0.90 (0.54 – 1.49)	0.679	0.90 (0.54 – 1.52)	0.703
	Obese	47	0.50 (0.24–1.01)	0.063	0.63 (0.29 – 1.31)	0.227	0.64 (0.29 -1.35)	0.251

*Legend*: *INR* Indian Rupee. Adequate dietary diversity (dietary diversity score ≥ 5). Middle school certificate (8 years of education), higher secondary certificate (12 years of education)

*Two unemployed family members are included in this category

^**$**^Multivariate logistic regression model includes all covariates in the table, age of participants, and religion

^**$$**^Multivariate logistic regression model includes all covariates in the table, age of participants, religion, and energy intake

## Discussion

Among pregnant slum-dwelling women from Pune, India, more than half of the women consumed lower amounts of energy and protein than the EAR. Thirty nine percent of women exceeded the recommended carbohydrate intake (as E%), whereas protein intake (as E%) was within the recommended range for all women. Diets of pregnant slum dwellers were lacking in most micronutrients, especially iron, zinc, riboflavin, thiamine, and folate. Only one in three women had adequate dietary diversity. All women consumed starchy staples, approximately 80% consumed pulses and legumes, and 60% consumed other vegetables. Higher educational and occupational status of the primary earning member of the family and lower parity of women were related to having adequate dietary diversity.

### Comparison with other Indian and international studies

Our results on nutrient intake are in line with previous studies on macro- and micronutrient intake and deficiencies from India and other South Asian countries [1, 5, 35]. The average energy and protein intakes in our study were similar to that reported in the national nutrition report of urban India that was conducted at household level [36] and also in a national survey among pregnant women [35]. A study conducted among pregnant women living in Delhi slums a decade ago showed suboptimal consumption of protein and important minerals and vitamins by nearly half of the women [37]. However, our participants had a higher proportion of women consuming diets lacking in micronutrients. It has been observed that the average Indian diet has not changed much over the years to overcome the major gaps in intakes of micronutrient-rich foods [38].

Women with adequate dietary diversity consumed diet with cereal-based staples, pulses, other vegetables, milk and milk products and other fruits. Our study reported similar mean DDS as the studies conducted among pregnant women from different parts of rural India (mean DDS ranging between 4–5); however, the percentages of women reporting adequate dietary diversity was lower in our study compared to the studies conducted in rural India [22–25]. Farming land ownership, kitchen garden, possession of milk producing animals may facilitate the higher percentage of women with adequate dietary diversity in rural India. On the other hand, easy availability, and affordability of unhealthy foods (sugars, fats, highly processed foods) might be the reason for a lower percentage of women with adequate dietary diversity among pregnant slum dwellers in our study as these foods do not contribute to the composition of the DDS [9] reflecting ‘nutritional transition’ phenomenon [39]. The mean DDS in our study was similar, and the proportion of women with adequate dietary diversity was at least 15% higher in studies in Nepal and Eastern Ethiopia (and similar in Bangladesh) than in our study [40–42]. These discrepancies could be explained by differences in geographical locations, the tools and food items used to measure dietary diversity, the trimester in which the dietary data were collected, and the interviewing techniques. Importantly, the discrepancies could be because the current study participants were slum dwellers, their socioeconomic status may not be comparable to that of the women in other studies, and this may have impacted their dietary diversity. In our study, the group with inadequate dietary diversity had similar fat intake and higher carbohydrate intake than the adequate dietary diversity group. These findings are in line with the observations that a higher percentage of women consumed sweet snacks or sweet beverages in the inadequate dietary diversity group than that in the adequate dietary diversity group but the percentage of women consuming fried and salty foods were similar in two groups.

Higher educational and occupational status of the primary earning member of the family and lower maternal parity were associated with adequate dietary diversity. The primary earning members were either the husbands or the in-laws of the pregnant women. In joint families in the Indian society, these members have the authority to make decisions in the household including health seeking behaviour decisions. Thus, the higher education and occupation level of the primary earning member may have positively impacted the diet quality of the pregnant woman. Similar findings are reported from other studies from India, Bangladesh, and Ethiopia that the occupation of the husband or the head of the family influenced the dietary diversity of the pregnant woman [22, 41, 42]. However, women’s income and education were positively associated with higher dietary diversity in a study in Australia [43]. This difference could be due to the differences in the social structure of families between developing and developed countries. Additionally, similar to our findings, parity has been found to be inversely associated with diet quality in the USA and in Canada [44–46].

A community-based, multicity study spanning preconception through the delivery period and also including different aspects of food environment would shed more light on dietary habits among pregnant slum-dwelling women. As the Indian family is a complex and dynamic institution where decisions like food choices are dependent on the husband or the in-laws [47], future interventions may focus on involving family members to improve the dietary quality of the pregnant slum dwellers. Since low dietary diversity also reflects poor household food security [48], interventions may include recommending healthier food options using cheaper and easily available local and seasonal foods to improve food security among pregnant slum-dwelling women.

### Strengths and weaknesses

Despite the widespread population of pregnant slum-dwelling women, their dietary intakes have been sparsely studied in the Indian context. Considering that slum dwellers collectively share environmental risks and interventions that work among urban non-slum population are not directly transferable to the slum-dwellers [49], The findings of this study have a great potential for supporting the development of tailored interventions focusing on improving dietary intake among pregnant slum-dwelling women. To obtain high quality data, the research staff was trained well, and the data were collected using standardised and validated methods. We adjusted the nutrient intake for total energy intake using the residual method, which provides a measure of diet quality and is reported to be useful in mitigating the effects of measurement errors occurring in self-reported dietary assessment. The nutrient intake estimates derived using the residual method are uncorrelated with total energy intake and are directly related to overall variations in food choice and composition [33]. We studied dietary diversity which is extensively validated against dietary quality in terms of nutrient adequacy and a well-suited tool in population level assessment in resource poor settings [50]. We also collected data on unhealthy food groups so that it could shed light on different forms of malnutrition.

The study has some limitations. First, this study was only conducted in one Indian city, which may limit its generalizability. However, the macro- and micronutrient intakes in our study were comparable to those in a national study [35]. Additionally, we recruited women attending an ANC clinic providing free care. Thus, it is possible that slum dwellers who did not attend these clinics are not represented in this group. The dietary data were self-reported and susceptible to reporting bias. However, the use of the multiple-pass dietary recall method along with food models ensured effective diet data collection. Furthermore, we have identified and presented food choices; however, this study did not capture the reasons for these choices as that was out of the study scope. As the study was conducted among slum-dwelling women, social desirability bias may also have influenced the reporting, resulting in overreporting of dietary intake. We reported dietary intake of nutrients, and this does not include intake from supplements. All women visiting ANC clinics are provided iron and folic acid and calcium tablets. However, compliance with the supplementation is a key issue, and only half of the pregnant women reported taking the tablets for at least 100 days based on the latest national survey [18]. We presented the dietary intake cross-sectionally in mid-pregnancy, which might not be representative of intake in early-pregnancy period due to nausea and vomiting which are common in early-pregnancy. Therefore, the nutrient intake levels might be even lower in early pregnancy. The mid-pregnancy follow-up rate of our study was hampered due to the COVID-19 pandemic, as many of the families shifted to their native places. This might introduce some selection bias in our study. However, the women presented in this study and those who were lost to follow-up had similar background characteristics at baseline.

## Conclusion

The diets of pregnant slum-dwelling women were found to be deficient in numerous important micronutrients. Two-thirds of women had inadequate dietary diversity and around sixty percent consumed at least two unhealthy foods indicating nutritional transition in this population. Tailoring nutrition counselling programs to the economic and social needs of slum dwellers is necessary to improve their health behaviours. Family involvement in counselling programmes could be one of the strategies to enable better compliance with good nutritional practices during pregnancy.

## Data Availability

The datasets cannot be made publicly available because public availability would compromise participant privacy. The datasets used and analysed during the current study are available from the corresponding author upon reasonable request.

## Abbreviations

BMI: Body Mass Index
DDS: Dietary Diversity Score
EAR: Estimated Average Requirement
SDG: Sustainable Development Goal
